# Successful Closure of a Tracheoesophageal Fistula Using an Over-The-Scope Clip

**DOI:** 10.7759/cureus.37577

**Published:** 2023-04-14

**Authors:** Osman Ali, Gurbani Singh, Sindhura Kolachana, Mohammed a Khan, Varun Kesar

**Affiliations:** 1 Department of Gastroenterology and Hepatology, University of Maryland Medical Center, Baltimore, USA; 2 Department of Gastroenterology and Hepatology, University of Maryland School of Medicine, Baltimore, USA; 3 Department of Gastroenterology and Hepatology, University of Maryland, Baltimore, USA; 4 Department of Gastroenterology, Virginia Tech Carilion School of Medicine, Roanoke, USA

**Keywords:** endoscopic approach, tef, diffuse large b cell lymphoma (dlbcl), over-the-scope clip, tracheoesophageal fistula

## Abstract

A tracheoesophageal fistula (TEF) is a pathological connection between the trachea and esophagus, which can either occur congenitally or be acquired. An acquired TEF may occur secondary to malignancy, chemoradiotherapy, infection, or trauma. Hallmark symptoms typically associated with TEF include choking with food intake, productive cough, pneumonia, or failure to thrive. The management of TEF has predominantly involved surgical or endoscopic intervention such as esophageal or airway stenting, suturing, or ablation. More recently, the endoscopic over-the-scope clip (OTSC) has emerged as an effective method of TEF management. The OTSC grasps the mucosa overlaying lesion and seals the defect, thus making it an effective treatment option for the endoscopic closure of various GI defects such as fistulas, bleeding ulcers, and perforations. We report a case of a TEF, acquired secondary to underlying malignancy, and its successful treatment with the use of an OTSC placement.

A 79-year-old female with a significant history of diffuse large B-cell lymphoma (DLBCL) currently undergoing chemotherapy was admitted to the hospital for aspiration pneumonia. She presented with persistent productive cough and subsequent limited oral intake ability while initially presenting for DLBCL six months prior with an enlarging right-sided neck mass. Her positron emission tomography-computed tomography (PET-CT) imaging showed a cavitary lesion in the superior mediastinum with increased fluorodeoxyglucose (FDG) lymphatic uptake. She had an esophagogram followed by an esophagogastroduodenoscopy (EGD), due to aspiration concerns, which demonstrated a fistula site with tracheal secretions about 20 cm from the incisors. An OTSC was used to close the esophageal opening and successful closure was confirmed using real-time fluoroscopic imaging by the unimpeded passage of contrast in the stomach without leakage. At follow-up, she was able to tolerate an oral diet without any significant difficulty or symptom recurrence.

We present a case of successful endoscopic management of TEF with an OTSC that resulted in immediate fistula closure and improvement in the patient’s quality of life. This particular case highlights the ability of OTSC to provide more durable and long-term closure than other management techniques due to its mechanism of grasping more tissue for approximation and its association with less morbidity compared to alternative surgical interventions. Although previous reports describing the technical feasibility and utility of OTSC in TEF repair support its use, there is still a paucity of data exploring the long-term efficacy of OTSC in TEF management; therefore, additional prospective studies are necessary.

## Introduction

A tracheoesophageal fistula (TEF) is a congenital or acquired pathological connection between the trachea and esophagus [1]. Congenital TEFs are often diagnosed immediately following birth or during infancy while acquired TEFs occur secondary to a variety of conditions, including chemoradiotherapy, malignant disease, post-intubation, infection, and trauma [1]. In adults, the majority of TEFs occur in the setting of esophageal or lung cancer. Typical symptoms are the inability to tolerate oral food intake, recurrent or persistent cough, and severe pneumonia [2]. Traditionally, TEFs were managed through surgical treatments; however, given the risks and technical difficulties associated with surgical management, minimally invasive endoscopic treatment has surfaced as the preferred method. Endoscopic approaches include fibrin glue injections, ablation, suturing, tracheal or esophageal stenting, and through-the-scope (TTS) clipping, which allow for closure of the fistula, relief of airway stenosis, and prevention of additional liquid or gas leakage [1,3,4]. Recently, the use of the over-the-scope clip (OTSC) system has emerged as a treatment option in the management of TEF [4], with only a series of case reports documenting its outcome and efficacy compared to other standard interventions. The OTSC system is a novel mechanical clipping device that allows for the endoscopic closure of GI defects, such as fistulas, leaks, and perforations, by firmly grasping the mucosa overlying the lesion and sealing the defect [4,5]. We report a complicated case presentation of a patient with TEF secondary to underlying malignancy, which was successfully managed with OTSC placement.

## Case presentation

**Figure 1:**
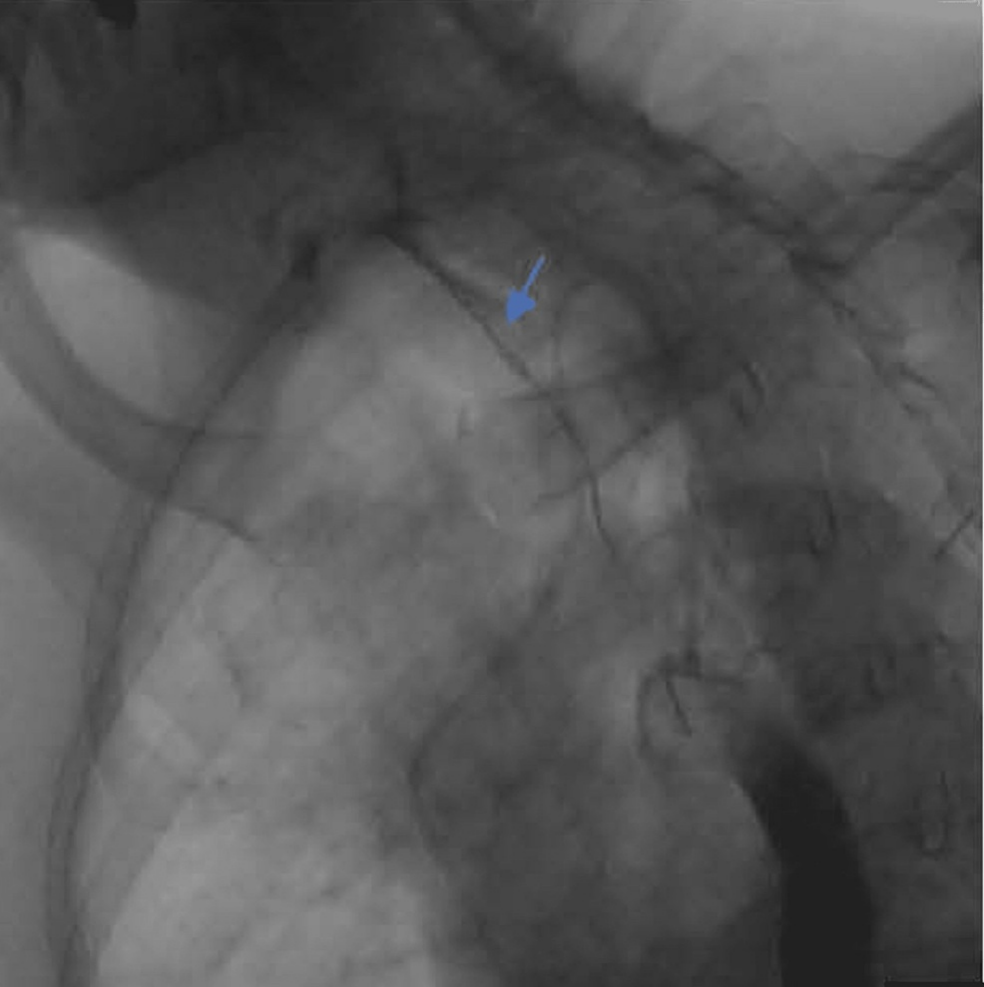
Esophagram revealing tracheal aspiration of contrast through a tracheoesophageal fistula (blue arrow) in the upper third of the esophagus

A 79-year-old female was re-admitted to the hospital for concerns of aspiration pneumonia after presenting with a persistent cough, excessive mucous production, and limited oral intake. She initially presented one month prior with a right-sided neck mass and was formally diagnosed with diffuse large B-cell lymphoma (DLBCL). Her positron emission tomography-computed tomography (PET-CT) imaging revealed a cavitary lesion in her superior mediastinum with F-fluorodeoxyglucose (FDG)-avid lymphadenopathy. During her most recent admission, she had an esophagogram due to concerns for aspiration (Figure 1, arrowhead), which revealed tracheal aspiration and a tracheoesophageal fistula in the upper third of the esophagus.

**Figure 2:**
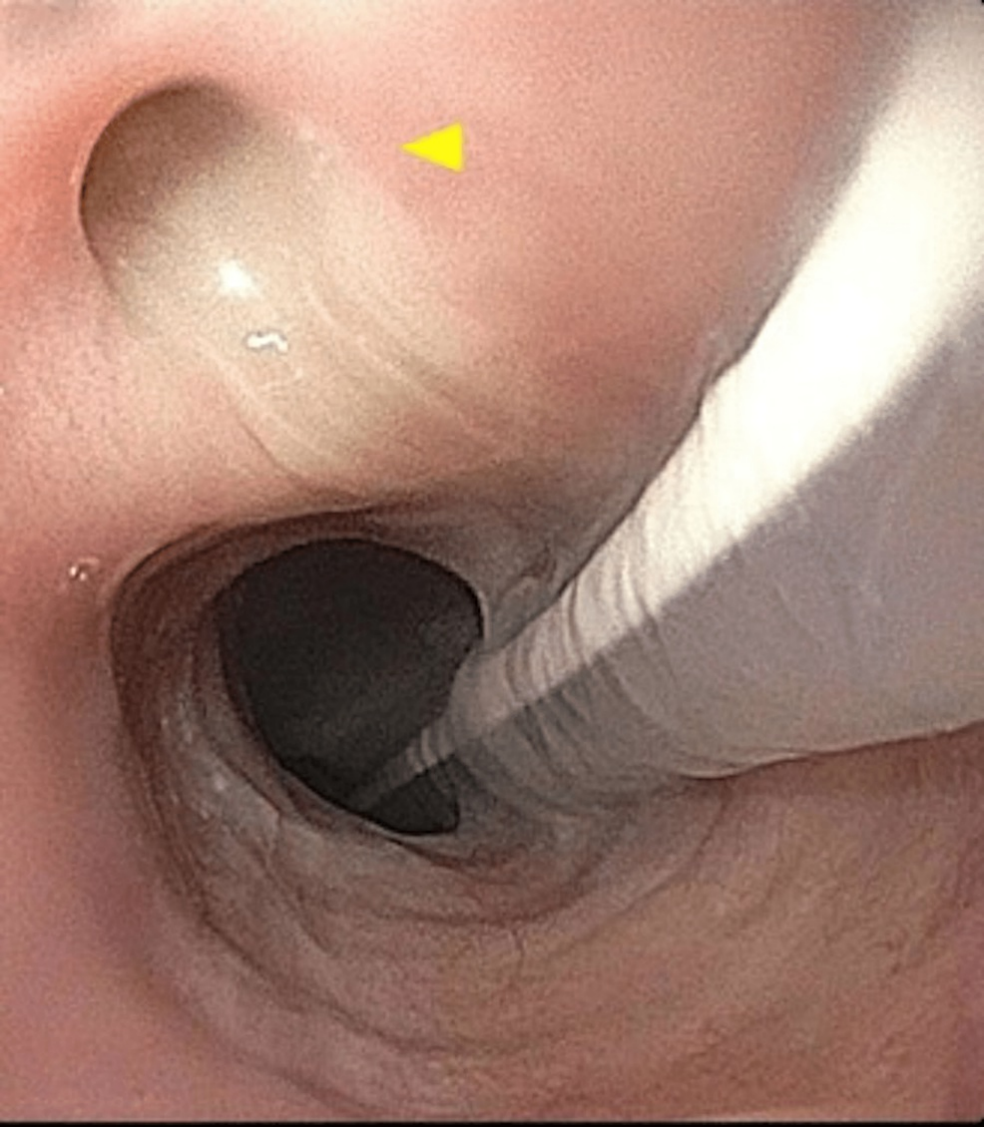
Direct endoscopic visualization of fistula site with tracheal secretions (yellow arrowhead) seen in the esophagus at 18 cm from incisors.

Upon these findings, an esophagogastroduodenoscopy was performed, which revealed a fistula site located 20 cm from the incisors (Figure 2, arrowhead), along with tracheal secretions.

**Figure 3:**
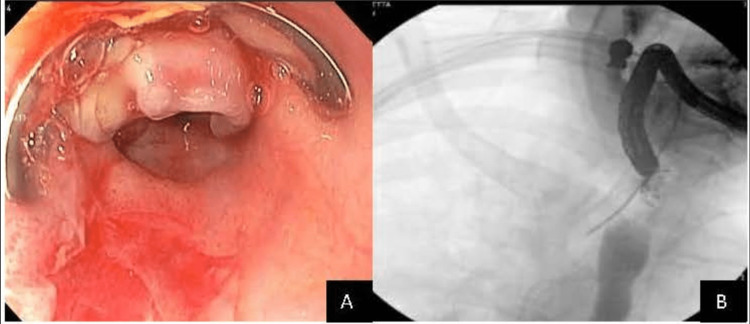
Endoscopic imaging (A) revealing an OTSC successfully closing the esophageal luminal fistula opening. Fluoroscopy imaging (B) confirming successful closure of the tracheoesophageal fistula communication and complete passage of contrast to the stomach OTSC: over-the-scope clip

Given the location of the fistula in the proximal esophagus (18 cm from incisors), it was determined that an over-the-scope clip (OTSC) would be preferred to the placement of a stent in order to prevent future stent migration. The OTSC successfully closed the luminal opening by approximating the adjacent tissue (Figure 3A). Further confirmation was achieved with fluoroscopy demonstrating the successful passage of contrast to the stomach without further leakage into the previously identified fistula (Figure 3B).

Following the procedure, the patient was advanced to a soft oral diet, which she tolerated without difficulty. Unfortunately, despite the closure of the fistula site, the patient succumbed to worsening hypoxia due to uncontrolled pneumonia and septic shock and was transitioned to comfort care per her wishes.

## Discussion

TEF is a pathological attachment between the esophagus and trachea and often is acquired secondary to malignancy, instrumentation, and trauma in adults [1,2]. Approximately 5-15% of TEFs occur in the setting of esophageal and bronchogenic carcinoma, and they can present clinically with dysphagia, coughing following oral intake, recurrent aspiration events, and poor nutritional status [3]. Due to the significant associated morbidity and mortality, appropriate management of TEFs is essential [6].

Management for TEFs is generally two-fold and aimed at treating both the underlying cause, as well as the physical fistula. Repair of TEFs can be achieved either surgically or endoscopically [3]. Surgical management of TEFs is usually reserved for larger fistulas and may be achieved through the placement of gastrostomy/jejunostomy feeding tubes, flap creation, or bypass surgery. However, endoscopic techniques are favorable for smaller fistulas or for individuals who are poor surgical candidates [3,6]. Specifically, individuals with TEFs in the setting of malignancy are better candidates for endoscopic techniques [7]. Endoscopic repair techniques vary depending on the size and location of the defect but include placement of stents within the trachea and/or esophagus, local occlusive therapies, and over-the-scope clipping (OTSC) [3,8]. Esophageal stents have been widely used to treat multiple esophageal pathologies, including strictures and fistulae in the setting of malignancy. Unfortunately, such stents are associated with complications such as stent migration, stent occlusion, and tissue ingrowth [9]. Specifically, stenting of pathologies located in the proximal esophagus, such as that observed in our patient, is frequently associated with stent migration [9,10]. OTSC is a novel procedure that allows for the endoscopic placement of clips, thereby facilitating the closure of various defects including variceal bleeds, anastomotic leaks, and fistulas [5]. Kobara et al. conducted a retrospective review of 1517 cases of various gastrointestinal diseases in which OTSC was applied. There was an average success rate of 78% for all pathologies ( i.e. bleeding, perforation, fistula, anastomotic dehiscence) with success measured through clinical endpoints such as radiographic evidence of resolution and the absence of complications [5].

Data regarding the efficacy of OTSC in TEF repair is limited; nevertheless, the system has been shown to have generally favorable efficacy. Within the aforementioned study by Kobara et al., the clinical success rate for fistula repair specifically was 52% (n = 388) [5]. Another study by Ramai et al. identified 10 cases of TEFs, all of which were successfully repaired with OTSC, and only one reported a complication of clip dislodgement identified on follow-up [8]. Multiple case reports have also demonstrated successful repair without long-term complications for TEFs of various etiologies. including malignancy, prolonged ventilation, extensive thoracic surgery, and congenital disease [11-14].

Although the literature describing the technical utility of OTSC in TEF repair is present, the long-term outcomes of this method are not well-defined. Complications associated with the general use of OTSC include intraluminal stenosis after placement of endoscopic clips, as well as laceration wounds and micro-perforations from the OTSC claws [5,15-17]. A retrospective chart review of patients who underwent OTSC for fistula closure found that nearly half experienced fistula recurrence by six months (n= 47). Notably, this study described gastrointestinal fistulas of variable etiologies, the most common being enterocutaneous and gastrogastric. Only one fistula in this study was a TEF, secondary to radiation therapy, and it was successfully treated with OTSC without the need for repeat intervention or the presence of fistula recurrence [15]. Another large retrospective study identified patients who underwent OTSC for fistula closure (n=91, n= 16 esophageal fistula). The researchers identified six technical failures, all of which were located in the upper gastrointestinal tract, and postulated that closure may have been complicated by fibrotic edges of the fistulae [18]. Multiple smaller retrospective studies have also attributed the failure of fistula closure to occur in the setting of fibrotic or necrotic edges of the lesions [11,12,19].

## Conclusions

Taken together, OTSC has been shown to be effective for the closure of gastrointestinal fistulae and, specifically, for TEFs. Although literature is limited, long-term complications of OTSC with respect to a TEF repair include intraluminal stenosis, fistula recurrence, and technical failure due to fibrosis of fistula edges. In this report, we present a unique case of successful closure of an upper esophageal TEF in the setting of diffuse B-cell lymphoma. OTSC may be useful for the treatment of TEFs and should be considered in the context of a given patient’s clinical presentation. Moving forward, long-term prospective studies are warranted.
